# Tuning Axial Resolution Independent of Lateral Resolution in a Computational Imaging System Using Bessel Speckles

**DOI:** 10.3390/mi13081347

**Published:** 2022-08-19

**Authors:** Vijayakumar Anand

**Affiliations:** 1 Institute of Physics, University of Tartu, 50411 Tartu, Estonia; vijayakumar.anand@ut.ee; 2 Optical Sciences Center, Swinburne University of Technology, Melbourne 3122, Australia

**Keywords:** Bessel beams, speckles, incoherent imaging, holography, diffractive optics, imaging

## Abstract

Speckle patterns are formed by random interferences of mutually coherent beams. While speckles are often considered as unwanted noise in many areas, they also formed the foundation for the development of numerous speckle-based imaging, holography, and sensing technologies. In the recent years, artificial speckle patterns have been generated with spatially incoherent sources using static and dynamic optical modulators for advanced imaging applications. In this report, a basic study has been carried out with Bessel distribution as the fundamental building block of the speckle pattern (i.e., speckle patterns formed by randomly interfering Bessel beams). In general, Bessel beams have a long focal depth, which in this scenario is counteracted by the increase in randomness enabling tunability of the axial resolution. As a direct imaging method could not be applied when there is more than one Bessel beam, an indirect computational imaging framework has been applied to study the imaging characteristics. This computational imaging process consists of three steps. In the first step, the point spread function (PSF) is calculated, which is the speckle pattern formed by the random interferences of Bessel beams. In the next step, the intensity distribution for an object is obtained by a convolution between the PSF and object function. The object information is reconstructed by processing the PSF and the object intensity distribution using non-linear reconstruction. In the computational imaging framework, the lateral resolution remained a constant, while the axial resolution improved when the randomness in the system was increased. Three-dimensional computational imaging with statistical averaging for different cases of randomness has been synthetically demonstrated for two test objects located at two different distances. The presented study will lead to a new generation of incoherent imaging technologies.

## 1. Introduction

The observation and study of speckle patterns dates back to the 20th century [1,2]. A speckle pattern is generated by an interference among mutually coherent optical fields with a random amplitude and phase commonly originating from a scattering object [3]. In the beginning, speckle patterns were considered as a problem to be minimized in imaging, and many speckle suppression techniques were developed to clean the images of the speckle noises [4,5,6]. Later, the randomness associated with the speckle patterns was found to be attractive for many applications such as interferometry, astronomical speckle imaging, optical lever, non-invasive imaging, super resolution imaging, biomedical imaging, and 3D sensing [7,8,9,10,11,12,13]. In recent years, artificial speckle patterns have been generated with spatially incoherent light sources using static as well as dynamic optical modulators for holography applications. The main difference between the speckles formed by a coherent and incoherent source originates from the coherence condition. In the case of a coherent source, the light from every object point is scattered and interferes with the scattered light from another point. With an incoherent source, the light from every object point is scattered, whose intensity is added to with the scattered intensity of another object point. Consequently, the process of addition in the case of an incoherent source reduces the visibility but allows the linearity condition in intensity which subsequently leads to a simpler imaging process [14,15].

The first such study of using speckles for indirect incoherent imaging was reported by Dicke and Ables in the year 1968, where a random pinhole array was used as the static optical modulator between the object and the sensor [16,17]. The indirect imaging method consists of three steps: calibration, imaging, and reconstruction. In the calibration step, the point spread function (PSF) of the system was recorded. The object intensity was recorded for a test object in the next step. In the final step, the image of the object was reconstructed by processing the PSF with the object intensity distribution in the computer. This computational reconstruction process can be as simple as a cross-correlation [18] or a highly complicated algorithm [19]. In recent years, speckles formed from incoherent light sources have been used for 3D (*x*, *y*, *z*) [20,21] and 4D (*x*, *y*, *z*, *λ*) [22] imaging applications. In many studies, the degree of chaos in the speckle pattern was controlled by controlling the scattering degree [23]. However, beyond that variation, there was no parameter that could be varied, and in the far-field, the speckle sizes of weak and strong diffusers become equal, resulting in a similar imaging behavior except for a slight change in the background noise. Therefore, the imaging characteristics of incoherent imaging systems based on speckles cannot be tailored beyond the limits of the direct lens-based imaging system, recalling that regular spatial correlation lengths are often diffraction limited [24]. In special cases, it is also possible to obtain correlations lengths beyond the diffraction limits by aperture engineering and the use of advanced reconstruction methods [24,25].

With developments in active optical modulators and the rapid developments in the area of imaging technologies, it is necessary to dissect the speckle theory further. In this study, for the first time, Bessel beams were used as the fundamental building block for the generation of speckle patterns. Bessel beams have many interesting characteristics such as self-healing, non-diffracting and, most importantly, a long focal depth [26,27,28,29,30,31,32,33,34,35]. Some recent developments on space-time vector light sheets exhibited a high resilience to scattering [36,37]. In this study, an object point is converted into a random array of interfering Bessel beams with different relative intensities and propagation directions. The sparsity of the interactions was varied and their imaging characteristics were studied. A synthetic computational 3D imaging experiment was carried out using two test objects for a few cases of sparsity conditions to demonstrate the variation in axial resolution. The manuscript consists of six sections. The theoretical analysis is presented in the next section. In the third section, simulation studies are presented. The synthetic computational 3D experiments are presented in the fourth section. In the fifth section, the experimental requirements are discussed. In the final section, conclusions and possible directions for future research are discussed.

## 2. Materials and Methods

**Figure 1:**
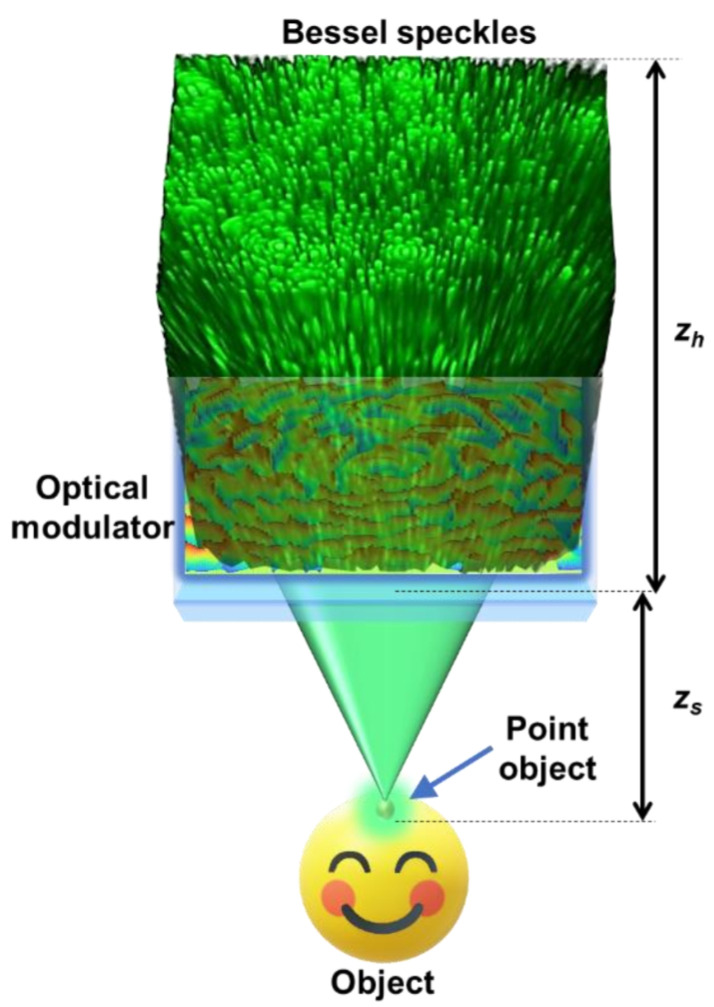
Optical configuration for generation of Bessel speckles.

The concept figure for the proposed study is shown in Figure 1. The light from an object point is converted by an optical modulator into a random array of interfering Bessel beams. The first step is to define and understand sparsity, which in this study is quantified by *σ* (which is the inverse of number of interfering Bessel beams *N*, when the number of beams is 1, *σ* = 1 and when the number of beams approaches a large number, *σ* → 0) [38]. When sparsity increases, the randomness decreases (and vice versa). In the first step, an optical modulator needs to be designed that can map every object point into a random array of interfering Bessel beams. There are different approaches to achieve this design. One direct approach involves the use of the Gerchberg–Saxton algorithm (GSA) with the spot arrangement in the spectral domain and a phase-only function at the space domain which can be calculated [38,39]. It is well-known that axicons in both refractive and diffractive versions can be used to generate Bessel beams [40]. As this is a theoretical study, the optical modulator was designed by summing diffractive axicon phases with different periods and linear phases. The complex amplitude of the optical modulator (OM) is given as

(1) $$\Psi_{OM}=\sum_{p=1}^{N}A_{p}\exp\left[-j2\pi \left(\frac{R}{\Lambda_{1p}}+\frac{x}{\Lambda_{2p}}+\frac{y}{\Lambda_{3p}}\right)\right]$$

where, $R=\sqrt{x^{2}+y^{2}}$ is the radial coordinate, Λ_1_, Λ_2_ and Λ_3_ are the periods of the axicon, *X* and *Y* components of the linear gratings respectively and *A* is the amplitude. The randomness of *A*, Λ_1_, Λ_2_ and Λ_3_ was controlled by an independent uniform random variable $C\cdot U\;\left[0,1\right]$, where *C* is a constant used to set limits for different parameters (namely amplitude and periods).

The light from a point object located at $\bar{r_{s}}=(x_{s},y_{s})$ with an amplitude of $\sqrt{I_{s}}$ reaches the optical modulator located at a distance of *z_s_*. The complex amplitude entering the optical modulator is given as $C_{1}\sqrt{I_{s}}L\left(\frac{\bar{r_{s}}}{z_{s}}\right)Q\left(\frac{1}{z_{s}}\right)$, where $Q\left(a\right)=exp\left[j\frac{\pi a}{\lambda }R^{2}\right]$ is a quadratic phase function and $L\left(\frac{\bar{s}}{u}\right)=exp\left[\frac{j2\pi \left(s_{x}x+s_{y}y\right)}{\lambda z_{s}}\right]$ is a linear phase function and *C*_1_ is a complex constant. The complex amplitude after the optical modulator is given as $C_{2}\sqrt{I_{s}}Q\left(\frac{1}{z_{s}}\right)L\left(\frac{\bar{r_{s}}}{z_{s}}\right)\Psi_{OM}$, where *C*_2_ is a complex constant. The PSF is observed at a distance of $z_{h}$ from the optical modulator which is given as

(2) $$I_{\mathrm{PSF}}\left(\bar{r_{0}};\;\bar{r_{s}},z_{s}\right)={\left|C_{2}\sqrt{I_{s}}Q\left(\frac{1}{z_{s}}\right)L\left(\frac{\bar{r_{s}}}{z_{s}}\right)\Psi_{OM}⊗Q\left(\frac{1}{z_{h}}\right)\right|}^{2}$$

where $\bar{r_{0}}$ is the location vector in the sensor plane and ‘⊗’ is a 2D convolutional operator. For an axicon, i.e., $\Psi_{OM}=\exp\left[-j2\pi \frac{R}{\Lambda_{1p}}\right]$, there is always an annular region in the axicon that satisfies the imaging condition of Equation (2) resulting in the central maximum in the sensor plane. The other regions of the axicon that do not satisfy the imaging condition form the rings around the central maximum. When the difference between the phase for the imaging condition and the phase of the annular region [41,42] is larger, the diameter of the ring is larger (and vice versa). When *z_s_* is varied in Equation (2), the central maximum is contributed by different annular regions of the axicon until it reaches a point when the phase of any region of axicon cannot satisfy the imaging condition. At this point, the boundary of the focal depth and the pattern starts to change into a ring pattern, becoming larger in diameter with distance henceforth. Within the focal depth, the Bessel beam exhibits a sharp central maximum and rings around it with decreasing intensity values of its radius. When the optical modulator consists of many axicons with different linear phases as shown in Equation (1), multiple Bessel fields *J*_0_ with different spatial frequencies, angles, and relative strengths were generated at different locations in the sensor plane. Wherever there is an overlap between the Bessel fields, there is self-interference. The intensity distribution at the sensor plane can be simplified as

(3) $$I_{\mathrm{PSF}}\left(\bar{r_{0}};\;\bar{r_{s}},z_{s}\right)=I_{\mathrm{PSF}}\left(\bar{r_{0}}-\frac{z_{h}}{z_{s}}\bar{r_{s}};0,z_{s}\right)$$

A 2D object can be considered as a collection of points represented as Kronecker Delta functions given as

(4) $$o\left(\;\bar{r_{s}}\right)=\sum_{p=1}^{M}a_{p}\delta \left(\bar{r}-\bar{r_{s,p}}\right),$$

where *M* is the number of points and *a* is the amplitude at every point. As the object is illuminated by a spatially incoherent light, the light diffracted from a point do not interfere with light diffracted from another point but their intensities add up. Therefore, the object intensity distribution is given as

(5) $$I_{O}\left(\bar{r_{0}};\;z_{s}\right)=\sum_{p=1}^{M}a_{p}I_{\mathrm{PSF}}\left(\bar{r_{0}}-\frac{z_{h}}{z_{s}}\bar{r_{s,p}};0,z_{s}\right)$$

The image of the object is reconstructed by a cross-correlation between *I_O_* and *I*_PSF_, given as

(6) $$I\left(\bar{r_{R}}\right)=\iint I_{O}\left(\bar{r_{0}};\;z_{s}\right)I_{\mathrm{PSF}}{}^{*}\left(\bar{r_{0}}-\bar{r_{R}};\;z_{s}\right)d\bar{r_{0}}\;=\iint \sum_{p=1}^{M}a_{p}I_{\mathrm{PSF}}\left(\bar{r_{0}}-\frac{z_{h}}{z_{s}}\bar{r_{s,p}};0,z_{s}\right)I_{\mathrm{PSF}}{}^{*}\left(\bar{r_{0}}-\bar{r_{R}};\;z_{s}\right)d\bar{r_{0}}\;=\sum_{p=1}^{M}a_{p}\Lambda \left(\bar{r_{R}}-\frac{z_{h}}{z_{s}}\bar{r_{s,p}}\right),$$

where Λ is the sampling function of the object similar to a Kronecker–Delta-like function. When the number of Bessel beams increases, then in addition to propagation, there are interactions between the Bessel beams resulting in self-interference distributions. While every Bessel beam is non-diffracting by nature, with an increase in number of Bessel beams, the interactions increase which changes the intensity distributions in the sensor plane with depth. These interactions vary randomly as the phase of different Bessel beams vary with the change in the location of the object point. A single Bessel beam may be applied for imaging application but with more than one Bessel beam, the object information becomes distorted in the sensor plane. Since the study is carried out using spatially incoherent and temporally coherent light, the system is linear in intensity. Therefore, it is possible to study the imaging characteristics in indirect imaging mode as in Equation (6). So, in the case of a single Bessel beam, the central maximum samples the object information faithfully, while the rings around the central maximum generate imaging noises. In this case and also the cases where the *I*_PSF_ is not a Delta-like function, an indirect imaging method needs to be applied. It has been identified recently that a non-linear reconstruction (NLR) method gives the highest SNR compared to matched filter, phase-only filter and Weiner filter [9,24]. The NLR is given as $I_{R}=\left|F^{-1}\left\{{\left|\tilde{I_{\mathrm{PSF}}}\right|}^{o}\exp\lceil j\cdot \arg\left(\tilde{I_{\mathrm{PSF}}}\right)\rceil {\left|{\tilde{I}}_{O}\right|}^{r}\exp\left[-j\cdot \arg\left({\tilde{I}}_{O}\right)\right]\right\}\right|$, where *o* and *r* are varied until the lowest reconstruction noise is obtained, arg(∙) refers to the phase and $\tilde{B}$ is the Fourier transform of *B*. So, in the indirect imaging framework for a single Bessel beam, the imaging PSF is not a Bessel distribution but a non-linear autocorrelation of a Bessel distribution which is a Kronecker Delta-like function [42].

## 3. Simulation Studies

**Figure 2:**
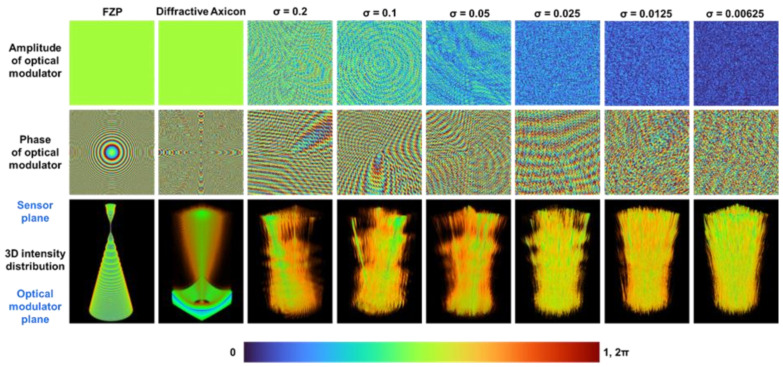
Amplitude, phase and axial intensity distribution for FZP, diffractive axicon (*σ* = 1), self-interfering Bessel beams *σ* = 0.2, 0.1, 0.05, 0.025, 0.0125, and 0.00625.

A simulation was carried out in MATLAB with a matrix size of 500 pixels along *x* and *y* directions, sampling size of 10 μm; a spatially incoherent and temporally coherent light source with a single wavelength λ = 632 nm is considered. For correlation based indirect incoherent imaging, a narrow spectral width is desirable, as a broad spectral width increases the width of the autocorrelation function and reduces the imaging resolution and signal to noise ratio [43]. For the simulation study, the sparsity values *σ* = 1, 0.2, 0.1, 0.05, 0.025, 0.0125, and 0.00625 are considered. As with direct lens-based imaging system, an indirect imaging system also has the same lateral and axial resolution limits given as ~λ/*NA* and ~λ/*NA*^2^ respectively, where *NA* is the numerical aperture *D*/*z*_s_ and *D* is the diameter of the entrance pupil which is 5 mm and *z_s_* and *z_h_* are 50 cm in this study. To have a reliable comparison, the simulation results are also compared with direct imaging using a Fresnel Zone Plate (FZP) with *z_s_* and *z_h_* in 2*f* configuration (*f* = 25 cm) and an axicon with a minimum period. The amplitude and phase of the optical modulators FZP, diffractive axicon (*σ* = 1) and optical modulators for generating self-interfering Bessel beams for *σ* = 0.2, 0.1, 0.05, 0.025, 0.0125, and 0.00625 and their axial distributions from the optical modulator to the sensor are shown in Figure 2. The axial distribution was calculated at every plane by varying *z_h_* in Equation (2) from the plane of the optical modulator to the sensor plane and the 2D information was accumulated into a cube data. As seen from Figure 2, with an increase in the sparsity *σ*, the density of the Bessel beams increases. The individual Bessel beam can be identified by the green lines while the side lobes and the interference effects are observed in orange color.

**Figure 3:**
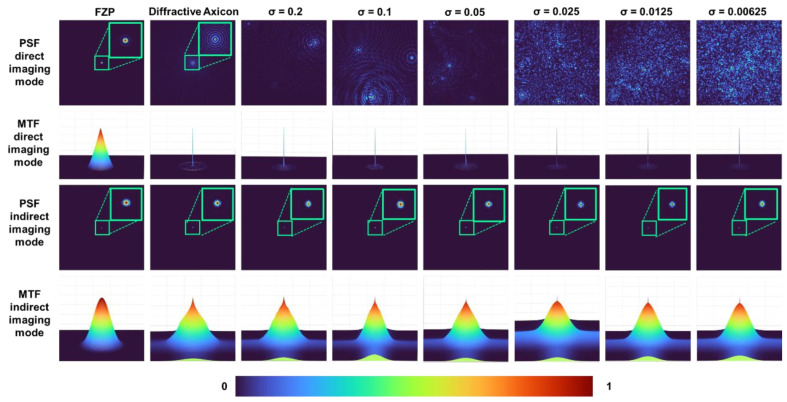
PSF and MTF obtained for direct and indirect imaging modes for FZP, diffractive axicon (*σ* = 1), self-interfering Bessel beams *σ* = 0.2, 0.1, 0.05, 0.025, 0.0125, and 0.00625. The MTFs are normalized to 1.

**Figure 4:**
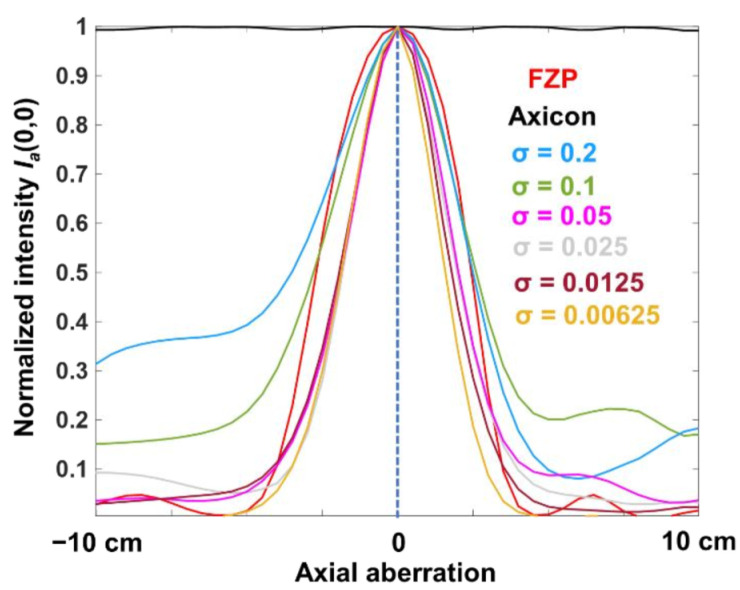
Plot of *I_a_* (0,0) with respect to Δ*z* for FZP, axicon and optical modulator with *σ* = 0.2, 0.1, 0.05, 0.025, 0.0125, and 0.00625.

The images of the PSFs for FZP, axicon and optical modulators with *σ* = 0.2, 0.1, 0.05, 0.025, 0.0125, and 0.00625 at the sensor plane located at 50 cm from the optical modulator and their corresponding modulation transfer functions (MTF) given as $\mathrm{MTF}=a\left|F\left(I_{\mathrm{PSF}}\right)\right|$, indirect PSFs obtained using NLR and their MTFs are shown in Figure 3. The optimal values of *o* and *r* for the least reconstruction noise quantified by entropy in this case was 0 and 0.6 respectively [24]. It is seen that in indirect imaging mode, the number of Bessel beams have almost no impact on the lateral resolution of imaging. This is an interesting result as the increase in randomness significantly changes the intensity distribution in direct imaging mode, while it is not varying in indirect imaging mode. The next important characteristic of an imaging system is its axial resolution. The axial resolution is the measure of how rapidly the intensity along the optical axis decreases when the object location is shifted away from the imaging condition. In indirect imaging, this can be measured using a cross-correlation between the intensity distribution corresponding to a particular plane in the object space and the one obtained corresponding to another plane and measuring the intensity value at the origin. The axial intensity variation curve can be obtained as $I_{a}=\left|F^{-1}\left\{{\left|\tilde{I_{\mathrm{PSF}}\left(\Delta z=0\right)}\right|}^{o}\exp\left[j\cdot \arg\left(\tilde{I_{\mathrm{PSF}}\left(\Delta z=0\right)}\right)\right]{\left|\tilde{I_{\mathrm{PSF}}}\left(\Delta z\right)\right|}^{r}\exp\left[-j\cdot \arg\left(\tilde{I_{\mathrm{PSF}}(\Delta z)}\right)\right]\right\}\right|$, where *Δz* is the shift in the location of the object in the object plane. The point object’s location was shifted from 25 cm to 75 cm and the axial curve was calculated for *σ* = 1 to 0.00625 as shown in Figure 4. It can be seen that with an increase in the number of Bessel beams, the focal depth decreases in the indirect imaging mode. This is an anomaly or non-linearity occurring only in the indirect imaging mode. In a direct imaging mode, the axial resolution and lateral resolution are intertwined and with a change in one affects the other. Let us consider a classical experiment of changing the aperture diameter. In this case, when the lateral resolution decreases, the axial resolution decreases (and vice versa). However, in indirect imaging mode, the lateral resolution is independent of the number of interfering Bessel beams while the axial resolution increases with an increase in the number of interfering Bessel beams. This is a unique behavior and will benefit imaging applications.

In the past, such hybridization was achieved by complicated holography experiments [44]. It must be noted that in all the reported imaging systems based on speckles such as interference-less coded aperture correlation holography [20], DiffuserCAM [19] and scatterplate microscope [21] such a non-linearity was not observed. In this case, the fundamental building block of the speckle pattern has been modified to introduce such a non-linearity where the lateral resolution is constant but the axial resolution varies for different cases.

## 4. Three-Dimensional Imaging

**Figure 5:**
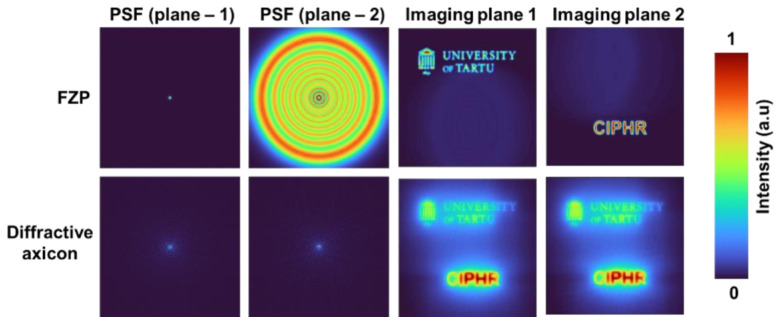
Images of PSFs and the imaging results of the two planes with two test objects for FZP and diffractive axicon.

A synthetic three-dimensional (3D) imaging is carried out next using two objects, namely “CIPHR” and “Tartu Emblem”, mounted at two different planes separated by a distance of 25 cm. As per the linearity condition in intensity, the object intensity distributions for object 1 and object 2 were simulated by a convolution operation with the corresponding PSFs. The resulting intensity distributions are summed to form the hologram. The object hologram can be mathematically constructed as $H=\overset{2}{\underset{q=1}{\sum }}\left|I_{\mathrm{PSF}}\left(z_{q}\right)⊗O_{q}\right|$. The energy for the two test objects was made equal, and so test object 2 appears weaker than test object 1 as the energy is distributed among many elements in test object 2. Recalling that any 2D object function can be expressed as a sum of sinusoidal spatial frequencies, larger features of object have smaller spatial frequencies and diffract light at smaller angles and vice versa. The numerical aperture of the imaging system restricts the information to a certain range of diffraction angles. Consequently, for larger features which predominantly diffract light at smaller angles, more energy reaches the sensor in comparison to smaller features. The above two effects contribute to the second test object being weaker than the first test object on the sensor plane. The PSFs corresponding two planes and the imaging results obtained by refocusing at the two planes for direct imaging with FZP and a diffractive axicon (σ = 1) are shown in Figure 5. As seen in the figures, the PSFs of a diffractive axicon remain unchangeable even with an axial error of 25 cm, while the PSFs of an FZP changes significantly. Consequently, the object information of one plane in the case of FZP is highly distorted, while in the case of diffractive axicon, due to a high focal depth the information from both planes are focused. However, the Bessel intensity distribution which is the reason for the high focal depth also distorts the information due to the ring pattern around the central maxima. Comparing the object information in the imaged planes, the object information obtained for FZP is sharper than that of diffractive axicon.

**Figure 6:**
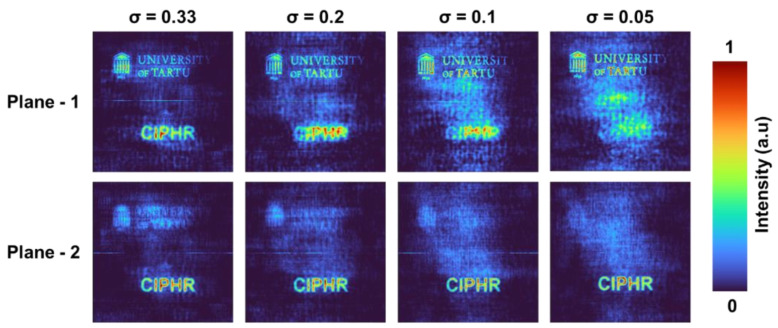
Reconstruction results obtained for two test objects located at two planes for *σ* = 0.33, 0.2, 0.1, and 0.05.

The reconstruction results at the two planes corresponding to the two PSFs with a spacing of 25 cm for *σ* = 0.33, 0.2, 0.1, and 0.05 are shown in Figure 6. There was significant background noise which was suppressed by a statistical averaging procedure involving mutually exclusive $\Psi_{OM}$ functions. In this study, three mutually exclusive samples were used for statistical averaging. It is seen that with a decrease in the sparsity, the axial resolution improves. All of the reconstructions of objects were carried out for *o* = 0 and *r* = 0.8 which is optimal in this case instead of the values *o* = 0 and *r* = 0.6 as in the case of PSFs. It is seen for σ = 0.2, the other plane information is quite visible in both reconstructions. As σ gradually decreases, the other plane information becomes less visible (which indicates the increase in the axial resolution).

## 5. Discussion

The implementation of the concept in an optical experiment requires a spatially incoherent light source such as a light emitting diode, an active optical modulator such as a spatial light modulator (SLM), an image sensor and controlling unit such as a computer to send data to SLM and acquire data from the image sensor. The light from the LED critically illuminates a pinhole using a refractive lens, and the light from the pinhole is polarized along the active axis of the SLM and the generated self-interfering Bessel beams are recorded by the image sensor. The pinhole is then shifted to different axial locations and the PSF library is recorded next which is then stored in the computer as the calibration dataset. An object is then placed within the axial boundaries of the PSF library and an object intensity pattern is recorded. The non-linear reconstruction is then implemented between the PSF library and the object intensity distribution, which reconstructs the object’s image corresponding to different axial planes. One of the challenges foreseen is the design of the phase-only mask for the generation of randomly self-interfering Bessel beams. Since this is a theoretical study, the optical modulator has been designed as a complex one which is difficult to implement in actual experiment. Therefore, in the experiment, the random multiplexing method reported in the previous studies is required [38]. As seen in the simulation, one of the challenges is the background noise which can be suppressed by recording multiple camera shots followed by a statistical averaging. Further studies are needed to evaluate the performance of the system for recording dynamic objects, as statistical averaging will impact the temporal resolution of the system. This study is limited to qualitative analysis of the dependency of axial resolution on the density of interfering Bessel beams. An intensive quantitative analysis is needed in the future in order to engineer an ensemble of Bessel beams of different numbers, relative intensities, radial and linear phase components to achieve a desired behavior.

## 6. Conclusions

Speckle patterns are formed by the random interferences of mutually coherent waves originating from diffusive surfaces. Speckles, once considered a noise to be suppressed have led to the development of numerous imaging, sensing, interferometry and holography technologies. In recent years, there have been numerous studies reported on the application of artificial speckles formed by spatially incoherent light for 3D (*x*, *y*, *z*), 4D (*x*, *y*, *z*, *λ*) and 5D (*x*, *y*, *z*, *λ*, *t*) imaging and holography applications [45,46,47,48]. In this study, for the first time, the fundamental building block of speckle pattern has been selected as a Bessel beam and the imaging characteristics have been studied in an indirect imaging framework. In a classical imaging system, the lateral and axial resolutions are dependent on one another, where it is almost impossible to vary one without affecting the other. In this study, where Bessel beams were randomly interfered in the indirect imaging framework, a non-linear behavior was observed. When the number of self-interfering Bessel beams was increased, the lateral resolution remained a constant, while the axial resolution increased. This is a useful behavior for imaging applications.

A synthetic 3D imaging has been carried out using two test objects following the principles of intensity linearity in imaging and a similar behavior was observed. There is a generation of background noise due to correlation between two positive intensity distributions. Usually, multiple camera shots were recorded for every event and either bipolar or complex distributions were generated to reduce the background noise [20,44] during correlation. In this simulation study, the background noise was suppressed using statistical averaging with intensity distributions formed from mutually exclusive aperture configurations which improves the signal to noise ratio by the factor $\sim \sqrt{g}$, where *g* is the number of samples. The background noise does not affect the lateral and axial resolutions of the system. This study represents a significant step towards generalizing the concepts of indirect imaging using spatially incoherent light. In this case, two parameters namely the non-diffraction parameter of Bessel beam and the accumulation of random phases associated with the Bessel beams compete against one another creating a possibility to tune the axial resolution of the system without altering the lateral resolution. While in this study, only axial coordinate has been considered, a similar effect is also expected along the spectral coordinate. Bessel beam’s intensity distribution remains constant with a change in wavelength. However, with the ability to counteract the spectral behavior with randomness, it is possible to control the spectral resolution of the system. In the same framework, the study of polychromatic Bessel beams will be another interesting direction. In summary, I believe that this study will lead to a new generation of incoherent imaging and holography technologies, where the ingredients of an ensemble of self-interfering beams can be engineered to achieve desired imaging characteristics.

## Data Availability

All of the data generated are presented within this manuscript.
